# Report of Pellagra in the Georgia Baptist Orphan Home

**Published:** 1914-12

**Authors:** George C. Trimble

**Affiliations:** East Point, Ga., Physician to Georgia Baptist Orphan Home


### REPORT OF PELLAGRA AND TREATMENT IN THE GEORGIA BAPTIST ORPHAN HOME, OF HAPEVILLE, GA.

By George C. Trimble, M. D., East Point, Ga., Physician to Georgia Baptist Orphan Home.

The Georgia Baptist Orphan Home, with forty-five children, was moved from Atlanta to Hapeville in 1899. Notwithstanding many have left the Home, either by being adopted or by arriving at the age for leaving to follow a livelihood for themselves, there are at present two hundred and thirty-four (234) children there.

The writer has had the pleasure of being its principal physician since it was located at the present place, and feels keenly the responsibility upon him.

This Home has children from every section of Georgia and coming as they do, from the unfortunante families whose homes have not been furnished with the actual necessities of life, we have to deal from the time of their admission, with a class of people who have been poorly nourished and who give negative family histories.

We have had from time to time the usual diseases, Typhoid, Scarlet Fever, Tuberculosis, etc., but not until the summer of 1911 did we have this disease appear, which, I may say, is giving us so much interest today, namely, Pellagra.

When my attention was called by a matron to some special children, to my surprise and horror, I found the typical Pellagra eruption on the feet, legs, hands, arms and around the neck, with fissures and sores in the mouth, emaciation and other symptoms characteristic of this disease.

During the summer of 1911 we had twenty-two well marked cases of Pellagra.

As to the recurrence of new cases during the summer of 1912, I can give no information, as I was not in attendance during that summer.

In the summer of 1913 we had seventy-five (75) cases of Pellagra. Forty-nine (49) boys and twenty-six (26) girls, with whom I shall now deal. The ages of the children were from five to twelve years.

It is impossible to go into a detailed family history, it would involve so much time to get the information and I do not deem it necessary to be included in this report.

Two (2) cases recurred in children who had the disease in 1911. Four of these children, representing two families, entered the Home with Pellagra and they had either a father or mother with the disease.

Twelve cases were children who had tubercular parents on one or both sides. So far as I have been able to diagnose, four of the twelve children have developed tuberculosis.

Twenty-four (24) cases were boys who had Pellagra and Hook Worm. Ten (10) cases were girls who had Pellagra and Hook Worm.

One girl had Pellagra, Hook Worm and Tape Worm. Eleven children left the Home while under treatment, to accept homes with relatives. I have no further record of these children.

I may class the sixty-four (64) cases under consideration, into two (2) classes.

Thirty (30) cases were of the first type of Pellagra; thirty-six (36) cases were of the second type.

The thirty-six (36) cases were typical eruptive cases, peeling of the feet, legs, hands, arms and around the neck, ulcers in mouth and were badly emaciated.

The thirty (30) cases were of the first or milder type, did not exfoliate, but had the typical symptoms of Pellagra, as ulcers in the mouth and anaemia, slight increase color of the hands and feet.

In other words, it looked like the headlines of disaster, for I believe the conditions of the extremeties are only reflex symptoms, or as I have said, headlines which land a positive diagnosis on the shore.

Gentlemen, I do not desire to draw out a discussion as to the etiology of Pellagra, but do allow me to digress slightly before going to the treatment.

We all understand there is a great difference of opinion. Let the opinion still dominate that has been handed down for generations by our best students and investigators who believe in the Maize influence.

We may follow up the researches of Sambon, the Captain of the Sand Fly; the studies of the Thompson-McFadden Pellagra Commission, and in a measure, they are all unsettled in opinion, but their followers are many.

Our Pathologists and Gastro-intestinal Specialists, as well as our own experience, tell us of the great disturbance of the digestive organs, the abnormal condition of both secreting and excreting organs in the way of a chemical toxin or other agents disturbing the natural nutriment of our organism.

I believe many articles of food of unsound products will increase the seriousness of the disease, if they are not the forerunners in cause.

I believe it is through the portal of digestion that influences or exciting causes, low vitality, malnutrition, coupled with the family predisposition, and often other undercurrents, as Hook Worm, Tape Worm and Tuberculosis, will produce the foundation of Pellagra.

There is a peculiar interest to me in these cases. We have five (5) dormitories. One for the baby boys; one for the second size boys, and one for the large field boys. One for the baby girls and second size girls together, and one, the main building for the large girls.

The forty-nine (49) boys who had Pellagra were all the second size boys from the same dormitory, which contained about sixty (60) boys. The twenty-six (26) girls were all the second size girls and from the dormitory that contained the second size girls and the baby girls.

So you understand no large boys and no large girls, no baby boys and no baby girls had Pellagra. I cannot account for this peculiar condition of affairs, I wish I could. Possibly there was a deficient diet in the shift at this age, that is, before the children were fed more liberally as they worked, and as their vocations demanded.

Or perhaps it was one of Captain Sambon's wandering flies that decided, as he passed over in his aeroplane, to puncture these special boys with the sting of Pellagra. Not being pleased with the savour left on his beak, he passed by and kissed the girls of the second cottage only, and left the venom of Pellagra upon their cheeks, continuing his flight, as the South winds cast his aeroplane homeward to join his mates in the spring riplets and sand bars. However this may be, we will turn to the treatment.

The general hygienic surroundings must be kept as good as possible, for we realize this is a day of preventive as well as curative work, especially in this disease.

The diet is one of the most important aids to restore as nearly as possible the digestive organs to their normal functions.

With proper dieting and corrective agents we shall see the clinical symptoms disappear, especially among children, and a general physical improvement follow. When this has been done, much has been accomplished for the patient, though we may not have uprooted the cause. But if there is a specific cause, we have greatly reduced its effect.

Since the development of Pellagra in the Home, special attention has been given to diversified selective diet and proper cooking. There is a matron at each table to see that the service is reasonable in quantity.

Prior to the invasion or manifestation of this disease, the Home used a great many shipped food products in their cooked form, especially the corn products, as flakes, etc. The meal for bread has always been made from home-ground corn, raised on the Home farm, with one exception. Twenty-five (25) bushels of shipped corn was purchased in the summer of 1911, about the time of the first outbreak of Pellagra. In this connection I consider it very necessary to know the date of the corn purchased, but the present book-keeper informs me she cannot find it on record.

Since the development of Pellagra in 1913, we have eliminated the corn products from all children except the large field boys, and they eat it sparingly.

Meals (changeable.)

Breakfast—Oatmeal, rice, butter, baker's bread or biscuit, milk, meat, eggs, fruit, and syrup (one pouring).

Dinner or lunch—All varieties of vegetables, soup, bread, stewed fruit, milk, and butter. In winter the supply vegetables is navy beans, cabbage, peas and so on.

Supper—Baker's bread, butter, milk, fruit, eggs, meat, potatoes of both kinds and syrup.

Each child has a tooth brush and is taught how to use it. Independent drinking founts have been advised. General bath two or three times a week.

The medical treatment is rather short and routine.

If ulcers in the mouth are extensive, I apply nitrate of silver occasionally, but a milder application as Argyrol two or three times a day will usually be sufficient.

I have found that Warner's Eczema Lotion, applied to the extremities, gives more comfort than any other local application. Another favorite prescription is sodii biborate, acid boric, sodii salacylate, each two drams, glycerine and alcohol, each one ounce and water q. s. six ounces, and apply twice a day.

I confined ten of the most serious cases in bed for several days with only preliminary or minor medical treatment and special diet. I believe the rest treatment is very important in this class of cases and will assist greatly in their recovery.

INTERNAL TREATMENT.

Castor oil was a favorite drug, calomel, in small doses, to some patients, but as a rule I do not advocate it.

If the child had diarrhoea I would give castor oil and follow for a few days with bismuth subnitrate three of four times a day.

Calcium sulphide, one-half grain before meal.

Potassium chlorate, two and one-half drams tr. ferri chlor, one half ounce. Ess. pepsin (F) q. s. ounces six, M-sig. Teaspoonful in water after meals. I pushed this treatment for several weeks and after a few days' rest, I returned to it. If any cases did not yield to this treatment, I would give Fowler's Solution and other tonics.

You will see the inclination of the prescriptions, just read, to follow the line of treatment formulated, as possibly some of you know, by Dr. J. C. Johnson, whom I thank for his visit and consultation, and the high value of his services.

I am also highly indebted to Dr. A. G. Fort for his services in the Hookworm finding and treatment.

I desire also to express my appreciation of Mrs. Cox, the nurse, for her assistance in the work.

In conclusion, my experience is that children yield to the treatment more readily than adults, and when we can reach them early, I believe many will be permanently cured and saved from becoming victims of a most dreaded disease.

As this is a disease that seems to distinctly manifest itself in the summer, I directed the nurse to keep a close watch on the Pellagra children. I directed her to return to the former internal treatment for one month or longer as a precaution, with the injunction to remember the diet and elimination.

Assisted by the nurse, I have recently examined all children remaining in the Home, and have found no local symptoms of the disease, though we may have an outbreak at an early date, especially if the table supply is cut down.

You may also remember the difficulty under which a doctor labors, as an Institution of this kind desires to run on as economical basis as possible.

Gentlemen, I do not claim for myself any specially new discoveries as to the cause or treatment of Pellagra, connected with my success in the treatment. I have followed, to some extent, the line suggested by others who have studied and written on this disease, I begun to realize that there had been so much written and said by eminent men and men of authority on the subject of Pellagra, I felt my inability to treat my subject as I desired. However, as a general practitioner with no great opportunity for research, I have given you as best I can my experience at the Georgia Baptist Orphans' Home.

